# Models of care for musculoskeletal health: a cross-sectional qualitative study of Australian stakeholders’ perspectives on relevance and standardised evaluation

**DOI:** 10.1186/s12913-015-1173-9

**Published:** 2015-11-16

**Authors:** Andrew M. Briggs, Joanne E. Jordan, Robyn Speerin, Matthew Jennings, Peter Bragge, Jason Chua, Helen Slater

**Affiliations:** School of Physiotherapy and Exercise Science, Curtin University, GPO Box U1987, Perth, Australia; HealthSense (Aust) Pty Ltd, Melbourne, Australia; New South Wales Agency for Clinical Innovation, PO Box 699, Chatswood, NSW 2057 Australia; Liverpool Hospital, South Western Sydney Local Health District, Locked bag 7103, Liverpool Business Centre, Liverpool, NSW 1871 Australia; BehaviourWorks Australia, Monash Sustainability Institute, 8 Scenic Boulevard, Monash University, Melbourne, VIC 3800 Australia; Department of Health, Government of Western Australia, PO Box 8172, Perth Business Centre, Perth, 6849 Australia

**Keywords:** Policy, Evaluation, Model of care, System, Musculoskeletal, Expert, Chronic disease

## Abstract

**Background:**

The prevalence and impact of musculoskeletal conditions are predicted to rapidly escalate in the coming decades. Effective strategies are required to minimise ‘evidence-practice’, ‘burden-policy’ and ‘burden-service’ gaps and optimise health system responsiveness for sustainable, best-practice healthcare. One mechanism by which evidence can be translated into practice and policy is through Models of Care (MoCs), which provide a blueprint for health services planning and delivery. While evidence supports the effectiveness of musculoskeletal MoCs for improving health outcomes and system efficiencies, no standardised national approach to evaluation in terms of their ‘readiness’ for implementation and ‘success’ after implementation, is yet available. Further, the value assigned to MoCs by end users is uncertain. This qualitative study aimed to explore end users’ views on the relevance of musculoskeletal MoCs to their work and value of a standardised evaluation approach.

**Methods:**

A cross-sectional qualitative study was undertaken. Subject matter experts (SMEs) with health, policy and administration and consumer backgrounds were drawn from three Australian states. A semi-structured interview schedule was developed and piloted to explore perceptions about musculoskeletal MoCs including: i) aspects important to their work (or life, for consumers) ii) usefulness of standardised evaluation frameworks to judge ‘readiness’ and ‘success’ and iii) challenges associated with standardised evaluation. Verbatim transcripts were analysed by two researchers using a grounded theory approach to derive key themes.

**Results:**

Twenty-seven SMEs (*n* = 19; 70.4 % female) including five (18.5 %) consumers participated in the study. MoCs were perceived as critical for influencing and initiating changes to best-practice healthcare planning and delivery and providing practical guidance on how to implement and evaluate services. A ‘readiness’ evaluation framework assessing whether critical components across the health system had been considered *prior* to implementation was strongly supported, while ‘success’ was perceived as an already familiar evaluation concept. Perceived challenges associated with standardised evaluation included identifying, defining and measuring key ‘readiness’ and ‘success’ indicators; impacts of systems and context changes; cost; meaningful stakeholder consultation and developing a widely applicable framework.

**Conclusions:**

A standardised evaluation framework that includes a strong focus on ‘readiness’ is important to ensure successful and sustainable implementation of musculoskeletal MoCs.

**Electronic supplementary material:**

The online version of this article (doi:10.1186/s12913-015-1173-9) contains supplementary material, which is available to authorized users.

## Background

Australians enjoy one of the most accessible and high-quality healthcare systems in the world when benchmarked against other Organization for Economic Cooperation and Development member nations [1, 2]. Like other developed and, increasingly developing nations, health delivery systems and health policy makers are faced with the challenge of adapting to the changing needs of its consumers [3]. Population health profiles are shifting healthcare priorities from acute care needs and communicable diseases to chronic, non-communicable conditions due to their increasing prevalence [4], particularly musculoskeletal conditions [5]. The system is also challenged with an active reform agenda for primary and hospital-based care that includes changes to funding allocations, policy and governance and workforce roles [6].

In Australia, the morbidity and mortality burden imposed by chronic musculoskeletal conditions is second only to cancer [7]. The prevalence and impact of musculoskeletal conditions are projected to soar in Australia and other developed nations [4, 8–11]. Furthermore, across all areas of healthcare, including musculoskeletal healthcare, there is a failure in the translation of evidence on best practice from research into patient care [12–15]. Addressing these issues requires research evidence to articulate more efficiently with policy and practice to minimise the ‘evidence-practice’, ‘burden-policy’ and ‘burden-service’ gaps. Further changes required include health service care delivery; the manner in which health professionals are trained and practice; and the level of engagement by consumers in the management of their health [7]. These shifts are required to optimise health system responsiveness, sustainability and population physical and mental well-being.

Historically, musculoskeletal conditions have not been considered in a comprehensive manner in health policy for chronic disease management or primary prevention [16–19]. In recent years, this situation has been somewhat redressed, particularly in Australia, with the introduction of state-wide and national Models of Care (MoCs) for musculoskeletal health [7, 20] and other conditions. MoCs provide evidence-informed blueprints for the delivery of consumer-centred health services that are tailored to meet local operational requirements. As such, MoCs serve as a vehicle to drive evidence into policy and practice at a jurisdictional level. Although the structural components of a MoC necessarily vary according to its clinical or population focus, the core elements and information provided are based around the framework: *right care, right time, right team, right place, and right resources* [20]. Development of MoCs will also vary depending on the governance and health system processes in given nations. In Australia, a Health Network model is used to develop MoCs where cross-discipline stakeholders (including consumers and carers) are connected to discuss, debate and recommend consumer-centred strategies to address health issues [20]. A web of evidence is developing to substantiate the effectiveness of the implementation of specific musculoskeletal MoCs, as reported in our recent international review [21], as well as uptake at a jurisdictional level [22–25].

There remains no standardised national approach to the evaluation of musculoskeletal MoCs despite evidence to support their effectiveness in improving consumer outcomes and system efficiencies, and indications that they continue to evolve and be promoted in Australia [7, 26] and internationally [27]. Furthermore, there is a dearth of information in relation to evaluating MoCs ‘readiness’ for implementation as well as indicators of successful implementation. Here, we broadly define ‘readiness’ as the content and development processes required to ensure implementation success and sustainability, while ‘success’ is broadly defined as the outcomes achieved after implementation. There is also limited data on whether Australian end-users assign value to musculoskeletal MoCs in their work or to a standardised evaluation approach. The aim of this study was to explore end users’ views on the relevance of musculoskeletal MoCs to their work and value of a standardised evaluation approach.

## Methods

### Design

Cross-sectional qualitative study, which is reported according to the COREQ-32 checklist (refer to Additional file 1) [28].

### Sampling strategy

Subject matter experts (SMEs) across broad health, policy and consumer backgrounds were sampled for this study, consistent with a recently published framework [29]. SMEs were identified based on their established role(s) in the development or implementation of MoCs. Identification was made through established musculoskeletal networks, clinical leads of musculoskeletal service and research programs, and policy makers who have been involved in development or implementation of musculoskeletal MoC, as we have reported previously [20]. SMEs were sampled across three Australian states: Western Australia (WA), New South Wales (NSW) and Victoria (VIC). These states currently have the most active policy, multidisciplinary engagement in musculoskeletal healthcare, and health services research activity [7]. We also used snowballing methods to identify additional SMEs, not necessarily known to the research team, and who met the inclusion criteria. To ensure national representation and adequate heterogeneity of skill sets, inclusion criteria and a minimum sample size for each discipline group, were defined *a priori* by the research team (Table 1).

**Table 1:** Inclusion criteria for subject matter experts. SMEs identified in each discipline were required to meet all the inclusion criteria

	SME discipline (*a priori* minimum sample size)			
	Health policy/strategy or advocacy (n ≥5)	Clinical practice and/or clinical/health services research (n ≥6)	Health service or program delivery (n ≥5)	Consumers (inclusive of carers) (n ≥5)
Inclusion Criteria	● At least one SME per state of WA, VIC, NSW ● At least one SME reflecting each of the sub-categories below ● Experience at a senior^a^ level in musculoskeletal and/or chronic disease (that implicitly includes musculoskeletal health) health policy or advocacy for at least 1 year ● Awareness of Australian musculoskeletal MoCs as defined by Briggs et al. [7]	● At least one SME per state of WA, VIC, NSW ● At least one SME reflecting each of the sub-categories below ● Experience in delivery of clinical care for people with musculoskeletal conditions at a senior practitioner level^b^ for at least 5 years; or at least 5 years experience in undertaking clinical and/or health services research in musculoskeletal health at a senior level^b^ with evidence of peer-reviewed publication(s) in the area ● Awareness of Australian musculoskeletal MoCs as defined by Briggs et al. [7]	● At least one SME per state of WA, VIC, NSW ● At least one SME reflecting each of the sub-categories below ● Experience in health service or program delivery, coordination, management or funding related to musculoskeletal healthcare and/or chronic disease (that implicitly includes musculoskeletal health) for at least 1 year at a senior level^c^ ● Awareness of Australian musculoskeletal MoCs as defined by Briggs et al. [7]	● At least one SME per state of WA, VIC, NSW ● Person lives with a chronic musculoskeletal condition (at least 5 years) or cares for someone with a chronic musculoskeletal condition (at least 5 years) ● Awareness of Australian musculoskeletal MoCs as defined by Briggs et al. [7]
Sub-categories	● State or federal government health policy, strategy or MoC development ● State or federal government policy, strategy or MoC evaluation ● State or federal government health workforce policy or strategy ● National-level advocacy for musculoskeletal healthcare	Clinical disciples represented ● Physiotherapy ● Rheumatology ● General practice ● Endocrinology ● Pain or rehabilitation medicine ● Community pharmacy	● Private health insurance industry ● Health service management or coordination at tertiary setting ● Health service management or coordination at primary care setting	● At least one female ● At least one male

*SME* subject matter expert

*MoC* model of care

^a^at least senior officer or manager level of employment

^b^fellowship level for medical practitioners (e.g. FRACP, FRACGP); senior clinician level for other disciplines; associate professor level for researchers

^c^at least at the manager or head of department level

SMEs were invited to participate by personal email from the research team. The email invitation provided a hyperlink to a secure portal, powered by Qualtrics™ software (Provo, Utah, USA). At this portal, SMEs were provided with a background to the study and invited to consent to, or decline, the invitation to participate. On accepting the invitation to participate, SMEs were required to answer screening questions to determine eligibility and to collect demographic data. SMEs who declined the invitation were invited to provide a reason for their decision and to nominate other SMEs, facilitating snowballing recruitment.

### Ethics, consent and permissions

The Human Research Ethics Committee at Curtin University approved this study (RDHS-08-14) and all participants provided informed consent, including consent to report de-identified participant-level data.

### Development of interview schedule

Our multidisciplinary research team iteratively developed a semi-structured interview schedule to explore SMEs perceptions about:

i) aspects of musculoskeletal MoCs important to their work (or life, for consumers); ii) the usefulness of standardised evaluation frameworks to judge ‘readiness’ of a MoC prior to its implementation and successful implementation of a MoC iii) challenges associated with evaluating MoCs pre and post-implementation.

A draft interview schedule was pilot tested with eight SMEs who met the inclusion criteria (different from those included in this study). Two from each stakeholder group were interviewed using a cognitive interviewing approach to determine whether the questions were generating the anticipated information [30]. A probing paradigm was adopted where the interviewer (JEJ) asked direct questions to assess comprehension of each question, understanding of terminology used, and whether it was easy or hard to answer. To allow for refinement of questions, two rounds of four interviews were conducted 1 week apart. Based on feedback from the first round of piloting, a separate consumer interview schedule was developed. The consumer schedule was shorter than the original and re-worded to explore the same concepts using consumer-centred language. The second round of interviews did not provide any further insights in relation to comprehension of questions, and therefore no further pilot interviews were undertaken.

### Data collection

Data were collected in April 2015 by four interviewers experienced in qualitative methods (AMB, HS, MJ, RS). The interviewers had a broad range of career skills including clinical practice in musculoskeletal healthcare (AMB, HS, MJ), clinical practice across chronic diseases (RS), health service management experience (MJ), education (HS), health services research (AMB, HS, RS), health policy (AMB, RS) and MoC development, implementation and evaluation (AMB, HS, MJ, RS). Given cross-sector and cross-discipline experience of the interviewers and the sampling strategy used, some of the SMEs were known to the interviewers. In this context, the possible threat of responder bias was unavoidable since we identified *a priori* that a minimum level of contextual experience among SMEs was needed in order to provide meaningful data. To minimise any risk of dependent relationships, interviewers only interviewed SMEs where no direct reporting, working, personal, or financial relationship existed. To ensure consistency between interviewers, a senior qualitative researcher (JEJ) provided training to the interviewer team. Specifically this involved tailoring interviews to the knowledge and experience of interviewees, use of specified probing questions, and paraphrasing answers to confirm understanding [31]. Interviews were conducted by telephone and audio-recorded.

### Data analysis

Interviews were transcribed verbatim and analysed independently by two researchers: one a qualitative methods expert (JEJ) and the other a content expert (AMB). An inductive analysis based on grounded theory was used [32]. All transcripts were initially reviewed and codes derived from the data. Through an iterative process of reviewing transcripts and generating codes, the codes were further refined, grouped into concepts where similarities amongst codes existed, and key themes developed. Where discordance between analysts was identified, and where appropriate to gain consensus, these were discussed and transcripts re-reviewed. Descriptive statistics were used to characterise the SMEs.

## Results

Twenty-seven SMEs (*n* = 19; 70.4 % female) participated in the study across three states (WA: 12; NSW: 10; VIC: 5). Interview duration ranged from 18 to 52 min. Five (18.5 %) of the 27 SMEs were consumers. Three (11.1 %) SMEs reported some awareness of the Australian musculoskeletal MoCs, 10 (37.0 %) reported having reviewed at least one MoC, and 14 (51.9 %) reported having been involved in the development and/or implementation of at least one MoC. A summary of SME demographic characteristics is provided in Table 2.

**Table 2:** Demographic characteristics of the sample

SME discipline category	Current profession	N (%)^a^	Mean (SD) years in current professional role
			[range]
Health policy/strategy or advocacy	Health policy and/or program development for chronic disease	14 (51.9)	12.6 (6.5)
			[4–30]
	Public health system funding	4 (14.8)	6.3 (2.9)
			[4–10]
	Advocacy and/or consumer representation for musculoskeletal health	4 (14.8)	14 (12.4)
			[4–32]
	Health workforce policy/strategy	8 (29.6)	12.1 (6.6)
			[3–25]
Clinical practice and/or clinical/health services research	Clinical practice in musculoskeletal healthcare (currently active)	8 (29.6)	26.3 (4.7)
			[20–32]
	General practice	2 (7.4)	
	Endocrinology	1 (3.7)	
	Rheumatology	2 (7.4)	
	Pain/rehabilitation medicine	1 (3.7)	
	Community pharmacy	1 (3.7)	
	Physiotherapy	1 (3.7)	
	Clinical practice in musculoskeletal healthcare (currently inactive)		
	General practice	1 (3.7)	
	Physiotherapy	3 (11.1)	
	Clinical and/or health services research in musculoskeletal healthcare	4 (14.8)	21 (7.2)
			[15–29]
	Tertiary education of healthcare professionals	7 (25.9)	15.7 (8.3)
			[7–30]
Health service or program delivery	Health service delivery, coordination or management related to chronic diseases	10 (37.0)	14 (8.0)
			[5–30]
	Private health insurance	2 (7.4)	1.5 (0.7)
			[1-2]
Consumer	Consumer	5 (18.5)	31.8 (14.6)
			[15–50]
Other	Other (health economics; primary care system change and capacity building; development and evaluation of healthcare models	3 (11.1)	20.0 (5.0)
			[15–25]

^a^categories are not mutually exclusive, therefore the sum does not equal 27

Qualitative results are presented under the following headings:

1. Aspects of musculoskeletal MoCs that are important to stakeholders’ work.
2. Perceptions of standardised evaluation frameworks to judge ‘readiness’ and ‘success’ of musculoskeletal MoCs.
3. Challenges associated with evaluating musculoskeletal MoCs.

Where the term MoC is referred to, this relates to musculoskeletal MoCs.

### Aspects of musculoskeletal MoCs that are important to stakeholders’ work

MoCs were perceived as critical for providing a platform to influence and initiate changes to healthcare delivery, so as to align it with best-practice (see MoC as platform for change section), as well as providing practical guidance on how to implement and evaluate services (see MoC providing practical guidance to implement and evaluate services section).

“…we should all be evidence-based practitioners and we should be all trying to implement evidence into what we do, and so I see models of care and evaluation of Models of Care as an integral component of that” (SME 8)

#### MoC as platform for change

In considering MoCs as providing a platform for sector change, four critical information components were identified to imbue credibility in a MoC and increase the likelihood of uptake by stakeholders:

- the development and consultation processes
- a compelling case for change
- a clear outline of objectives, core elements and anticipated outcomes, along with performance indicators to evaluate outcomes
- a description of how services and resources would be delivered.

##### Inclusiveness in development of a MoC

The strongest theme to emerge was the need to demonstrate that a broad range of stakeholders had been consulted from inception of the MoC. This included representatives across the care continuum (i.e. primary through to tertiary sector) and different sectors (i.e. private, public, non-government, consumers, community).

“That the end product can demonstrate that its had the input from the right professions… it’s very much having a representative group…you know, multi-disciplinary, as well as having the people with influence, the colleges, the association, that kind of stuff. And involving them from the beginning. So, not just bringing them to review something at the end. It’s actually they’re involved in drafting and developing.” (SME 7)

Further, without endorsement from influential change champions and organisations, SMEs warned that MoCs can be perceived as incomprehensive, which would negatively affect engagement, or at worst, result in stakeholders being unaware of the MoC’s existence or ambivalent to it.

“Well I think the bad thing is, as a community pharmacist, a Model of Care is really not important to my work at all - it should be, but it’s not. And I think that’s because as a community pharmacist I had absolutely no awareness of [its] existence…” (SME 15)

##### Establishment of a case for change

A compelling case for change was also considered vital for uptake. This included a justification being based not only on best evidence, but also local data (jurisdictional/national), previous experiences and learnings and local expert opinion. Detailing the benefits of the MoC, how it would address ‘known’ local problems within the sector, and how proposed services would be integrated into existing service delivery models, were also perceived as essential.

“I would be interested in…the argument for the evidence in which the Models of Care are based and how that fits in the service delivery models both in terms of an economic perspective and also an integrated sort of model.” (SME 2)

“It’s got to be safe, so the safety elements have to be considered strongly. It must either improve or at least maintain the quality of care that’s already likely one would hope improve, but certainly not in any way diminish. It has to ensure that the effectiveness of care is going to be improved or again at least maintained…It should improve the patient journey and the patient experience. It should increase the efficiency from a service or system perspective.” (SME 5)

##### Providing a clear outline

Across disciplines, strong emphasis was placed on the MoC clearly defining its scope, objectives and outcomes. The language in the document needed to be meaningful to different stakeholders and to delineate between the core and non-essential components of the model. SMEs from primary care, in particular, stressed the need for simple, translatable strategies to assist implementation.

“One of the most important things is having a document which is actually going to be understandable to a person outside of that special [musculoskeletal] area…that specific managers understand it as well as clinicians [and] consumers.” (SME 13)

“And they’re [General Practitioners] not interested in pages of documentation, they’re not interested in the eighty page Model of Care document; they’re interested in two pages… Give it to me simply; have you got an assessment for me with my package that I can use, and how do I do it, and who do I refer to? Keep it really simple with a link to the more detailed document.” (SME 22)

##### Describing how services could be delivered

Detailing how musculoskeletal services would be delivered was of high importance to SMEs involved in providing health services and consumers. This included access and service components, as well as education strategies and support mechanisms for both health professionals and consumers.

“… as a clinician I think the most important things are that it [MoC] sets down…the way in which care will be delivered for people with musculoskeletal conditions…so that there’s hopefully a clear pathway with services available and accessible that need to be delivered in line with the model of care.” (SME 14)

Consumers specifically advocated that MoCs were important in facilitating the provision of accessible multidisciplinary services that are ideally located in the community. Examples included outreach and telehealth services. Consumers also identified the need for MoCs to include an emphasis on early identification and management, psychosocial health, and transition services for adolescents.

“I think probably the key point is access…consumers need to be able to access these opportunities easily with the minimal cost and reduced waiting times, and to be able to have a service that gives them as much multidisciplinary care as possible.” (SME 24)

#### MoC providing practical guidance to implement and evaluate services

The second key aspect of MoCs that was important to SMEs’ work was providing practical and detailed considerations for implementation and evaluation of changes to service delivery. A fundamental component was the demonstration of pilot testing across diverse sites to provide insight into outcomes and adaptability.

“I’d like to see whether a pilot study has been run in a small number of sites of different, maybe, locations and environments to make sure that the Model of Care is both effective, but is also generalizable.” (SME 12)

“…particularly when you see a preliminary evaluation– if there’s pilot studies done and we’ve, you know, had that advantage in New South Wales obviously to do some pilot studies and then present the results from the pilot studies, then I think that’s really important because they [stakeholders] say “Oh, I want that too…””(SME 20)

SMEs cited several practical components that were important to be included in a MoC to assist with implementation. This included identifying system requirements for implementation; detailing how a new MoC would replace an old or existing MoC; prioritising components for implementation and providing implementation plans or business cases. Similarly, guidance was also sought in relation to evaluation processes and identification of data sources to assist with evaluation.

“I think in advance it’s also helpful to make sure that the evaluation that you’d like to do at the end is thought about…so what on a day do you want to capture and systems that are required for that, and the person burden, but also resource burden in developing and implementing that…” (SME 10)

“…there’s a step between the Models of Care and then what it is that you plan to implement. The work we did…was trying to look at how you turn the Model of Care into discreet programs. I think there needs to be a lot more work of that nature. The real lesson…is that those processes then need rigorous project methodology and do need very structured implementation targets.” (SME 19)

### Perceptions of standardised evaluation frameworks to judge ‘readiness’ and ‘success’ of musculoskeletal MoCs

There was unequivocal endorsement for a framework that assessed whether critical components across the broader health system had been considered or were ready *prior* to implementation of an MoC; i.e. a ‘readiness’ framework (Evaluating the readiness of a MoC section). While evaluating the ‘success’ of an implemented MoC was considered important (Evaluating success of an implemented MoC section), this was perceived as an already familiar concept with a reasonably well established and understood approach, compared to an appraisal of ‘readiness’. The need for a structured approach to judging readiness resonated strongly with SMEs, based on their experiences with premature and unsuccessful implementation.

“I think an “evaluability assessment” or “readiness assessment”, whatever it’s called, is really, really important… [there have been] so many evaluations where you’ve gone in to do it and not only has it not been ready to implement, the enablers aren’t in place to support a new Model of Care, staff perceptions are quite obtuse, but there might not even be a baseline” (SME 4)

#### Evaluating the readiness of a MoC

A readiness evaluation framework was perceived as valuable in providing assurance to stakeholders that essential elements required for implementation are present in the target environment and that known barriers have been removed or worked through.

“…because it is difficult to know [when a MoC is ready] because so many parts of the system and such a wide variety of things need to align that it’s difficult to know when all of that stuff is aligning up…so some sort of framework or structured way or a model that could take you through…and give you the confidence that it’s now ready would be very valuable.” (SME 1)

The concept of readiness included assessing stakeholders’ willingness to change and whether the MoC was usable in the current environment. This was perceived as critical in building receptiveness towards the MoC.

“… it comes down to the whole principles of change management and whether you’ve got a receptive audience. Do you have fertile ground in which to plant this thing?” (SME 19)

“So I think it’s really important to make sure you’ve got everything, all the ingredients right, the context is right, that the people involved are ready and engaged with it, ready to deliver it and embrace it before you actually implement it and evaluate it.” (SME 8)

SMEs were also wary of any readiness evaluation framework being overly simplistic, and thus not capable of capturing the complexities of a dynamic healthcare environment or adapting across different environments. Similarly, SMEs cautioned that an evaluation framework should not be too prescriptive so that it became an obstacle to conducting meaningful evaluations.

“…in some ways I think there can be a tendency to boil everything down to a checklist, and that concerns me as well.” (SME 4)

“…it’s got to be seen as a value add exercise and not something that’s just kind of ticking a box to do a function that you know may or may not be useful.”(SME 14)

#### Evaluating success of an implemented MoC

SMEs strongly identified the need to distinguish between an evaluation of the implementation of a MoC (process evaluation) and an evaluation of the outcomes associated with the MoC itself (impact evaluation).

“…if you want to make statements or judgments about the effectiveness of a Model of Care, which presumably at some point you would want to because the whole – one of the purposes behind a Model of Care is to improve…and you can’t make judgments about effectiveness if you don’t know whether it’s been implemented appropriately in the first instance…” (SME 8)

“I guess we need to measure what we do to know whether we’re making a difference…I mean, we’re in a place of increasing need and demand, and we have finite resources, so…measuring what we do is important to make sure that it’s delivering value, so value in terms of quality and cost”. (SME 7)

Using a framework to inform a structured approach to exploring and documenting lessons learnt from implementation efforts was also deemed valuable. This provided opportunities for continued engagement with stakeholders and ongoing quality improvement for implementation efforts.

“I think measuring people’s commitment to the implementation and to the Model of Care is very telling so that’s really very important…how they’ve been engaged and enabled to change whatever it is that they have needed to change. That can be rich I think in terms of what you might do the next time, both good and bad…so what we might not do, but also but what you might want to replicate.” (SME 2)

“…evaluation is obviously also critically important for consumer satisfaction and potential need for modifying the Model of Care, assessing the consistency of uptake and obviously the acceptability to the stakeholders” (SME 9)

A standardised framework that facilitated evaluation of MoCs across sites, and even jurisdictions, was perceived as highly useful for the sector.

“…if there were a particular framework that could be used, then there is some measure of comparison rather than, well we evaluated this [MoC] implementation by this process but another evaluation process was used for [another] Model of Care…” (SME 3)

### Challenges associated with evaluating musculoskeletal MoCs

Whilst SMEs supported standardised evaluation frameworks to judge the readiness and success of MoCs, they were cognisant of the many challenges associated with this. Of these, the strongest themes to emerge were: (i) identifying, defining and measuring key indicators that adequately reflect ‘readiness’ and ‘success’ of a MoC (Identifying, defining and measuring indicators of readiness and success section) and (ii) recognising the potential impacts of systems or context changes within a dynamic healthcare environment on prospective evaluations (Impacts of systems or context changes on prospective evaluations section). Other challenges included system constraints, cost of evaluation, meaningful consultation with stakeholders and developing a framework that is applicable across diverse jurisdictions and healthcare settings (Other challenges section).

#### Identifying, defining and measuring indicators of readiness and success

While SMEs strongly indicated the need for an evaluation framework to be flexible and take into account different contexts and changing environments, they also identified perceived challenges. These included developing specific indicators that appropriately reflected readiness and success from MoCs and achieving consensus amongst stakeholders as to how these should be measured and defined.

“…Models of Care are pretty high level documents and as such they often lack that specificity or that operational level of detail and specificity of activities that allow for betterment, so it can be very difficult to know where to target your measures and determine what to measure. And then, yeah, there’s often a lack of agreement I think about how to measure, is something ready to be measured…” (SME 1)

SMEs identified that musculoskeletal conditions are highly diverse in their presentations and are very commonly co-morbid to other chronic health conditions. This complexity creates considerable heterogeneity in cohorts being evaluated and is a challenge to evaluation initiatives.

“ one [challenge] that I’ve also eluded to is the comorbidity - the fact that our patients are wonderfully diverse and that’s often our biggest dilemma is trying to decide whether somebody who’s got so many comorbidities is still worthwhile trying to include in it [MoC] or whether you know they are so different in all other respects and there only going to be a confounder.” [SME 11]

#### Impacts of systems or context changes on prospective evaluations

SMEs noted that the speed at which healthcare environments change could make evaluation difficult or render it out of date quickly. Risks were also perceived in attributing causation of observed outcomes to a MoC (either positively or negatively) given multiple influential factors present in any healthcare setting.

“… but the Model of Care is probably only one of the things that accounts for improved or changed outcomes in the patient population. Because almost invariably there are other things happening at the same time, some of them planned and some of them unplanned. So the evaluation framework around the Model of Care is very important but I think we have to also be a bit pragmatic and remind ourselves that sometimes when there is a change in outcomes, whether it’s good or bad, there is probably a number of factors impacting upon it and that’s always the dilemma…” (SME 11)

#### Other challenges

Of the several other challenges cited by SMEs relating to the development of an evaluation framework (Table 3), creating a framework that is applicable to different MoCs across jurisdictions was the most complex. This related not only to the range of different condition-specific musculoskeletal MoCs available and those likely to be developed, but also geographic variability in implementation sites such as urban and rural settings, and consequent variability in the availability of resources and workforce, modes of care delivery, and funding models.

**Table 3:** Other challenges associated with evaluating Models of Care

Key theme	Summary description	Illustrative quote
System constraints	Implementation of a MoC into an existing system may be unfeasible due to constraints within the current system. For example, some of the aspects of the MoC might need system enablers in place (e.g. new IT infrastructure), so implementation and subsequent evaluation cannot proceed successfully until system changes are completed. Additionally system design constraints, such as the split health funding models between the Australian Commonwealth and State/Territory governments, also presents as a significant barrier to evaluation across settings.	“So I think they’re all external constraints and it’s around the purchasing plan. So this is the amount of activity you will do and you know, this is the dollars that are attached to that because it’s worth you know, X number of dollars to – episode of care or service event and then it depends very much on the types of service models that the area health services or the local health network are wanting to implement. So depending on what the service models are, what the funding sources are, what the purchasing plans say, it’s really hard to do a pre and a post evaluation…”(SME 18)
Cost of evaluation	It was emphasised that evaluations can be resource intensive, depending on the study design, governance and data collection arrangements. SMEs indicated that external funding and partnership with Universities are ideally needed to assist with the collection of data (particularly an issue in the primary healthcare sector).	**“**One of the biggest issues we have in evaluating in general practice is that they don’t get paid for this sort of work…I actually get quite frustrated that it always ends up coming down to a dollar figure, but general practice isn’t paid to stop it’s work and to write an evaluation or to do a survey…one of the things that worked very well…they incentivised general practice to participate pre and post. It’s not a lot but to ensure that their nurse will be able to collect the information…” (SME 22)
Ensuring adequate involvement of stakeholders within the evaluation process	SMEs emphasised the need to ensure adequate involvement of stakeholders within evaluation processes in order to obtain a comprehensive understanding of issues relating to implementation and outcomes. Challenges in engaging stakeholders in evaluations included:	"I think quite often people jump to a solution and think they know the answer…we actually firstly need to have all the right people in the room, and when I say the right people, I don’t just mean the best clinicians, I also mean management of front line and I mean people who have a state wide role in funding and planning and some consumers.” (SME 16)
	● Ensuring all relevant stakeholders are involved, given diversity and complexity of healthcare settings relevant to musculoskeletal health, particularly in the private community setting.	
	● Getting stakeholders to understand the need to build evaluation into the entire process of a MoC; i.e. from inception to implementation.	
	● Achieving a cohesive understanding of terminology relating to MoCs across diverse stakeholders in different sectors of the care continuum.	

*SME* subject matter expert

*MoC* model of care

“…but national consistency and quality in a country like Australia, not just the [geographical] vastness, but the different jurisdictions, is always going to be problematic…” (SME 6)

## Discussion

### Main findings

Australian stakeholders in this study perceived MoCs to be critical enablers for system and service delivery reform and a blueprint for implementation and evaluation of new services. Our data are consistent with previously published reports, where MoCs have been identified as a mechanism to guide service planning and delivery for chronic health conditions [33–35], particularly musculoskeletal conditions [7, 21, 27, 36]. Importantly, we identified that MoCs hold different values to different stakeholders and that these value propositions could be enhanced and sustained by recognition of this diversity in the development of MoCs. Further, despite a range of challenges identified to achieving meaningful and valid evaluation outcomes, stakeholders agreed that an evaluation framework for musculoskeletal MoCs that included a strong focus on ‘readiness’ was important to ensure successful and sustainable implementation of musculoskeletal MoCs. While our focus was on musculoskeletal MoCs specifically, we suggest that our findings have relevance to MoCs for chronic diseases more broadly.

### Relevance of MoCs to stakeholders’ work

Stakeholders stressed the need for MoCs to be user-relevant and system-relevant, and that broad sector buy-in of the MoC was fundamental to achieving any meaningful level of sustainable implementation. Health and policy professionals cited the need for MoCs to adopt robust and comprehensive consultation methods and to clearly articulate a case for change and to identify performance indicators. In contrast, consumers identified the need for the MoC to address accessibility of services, particularly multidisciplinary care; consider support structures for families and adolescents; and directly address psychosocial health. These consumer-centred views on service planning and delivery align with other recent consumer reports [37–39] and highlight the importance of engaging these perspectives alongside other stakeholders. These different foci of relevance are reflective of the distinct ways in which these two stakeholder groups interact with the health system – one being a service administrator and delivery agent where processes, governance and adoption are important; and the other a consumer where point-of-care, access, scope and equity matter. Notably, a recent systematic review examining the effectiveness of chronic care models for health outcomes and health practices (for diabetes, cardiovascular disease, depression and chronic respiratory diseases), identified that of the 77 papers included in the review, only two included family support as part of the model, perhaps indicative of inadequate consumer input into the model design [33]. Further, another recent systematic review reported greater operational success in chronic care models when consumer perspectives were included [40].

Meaningful consultation, inclusive of a broad range of stakeholders, was considered an overall imperative in the development and planning implementation of a MoC. Inadequate or tokenistic engagement and consultation is a recognised barrier to uptake of programs, policies and models [41]. Australian MoCs, for example are generally developed through a network-based model of engagement, consultation and iterative implementation [20]. Such network-developed models are reported to be acceptable to stakeholders [26, 42], associated with improved outcomes for consumers [43], and identified as a facilitator to implementation of chronic care models [40]. Implicit within consultation, was the need for identification and engagement of clinical and administrative ‘change champions’ to support the development and implementation of a MoC; an established strategy for achieving knowledge translation into policy and practice [44–46]. Organisational champions have been similarly identified as important agents for facilitating organisational adoption of new programs or MoCs [33, 40, 47].

A compelling case for change was also identified as an important enabler for sector engagement. Bleser and colleagues reported that providing evidence-informed arguments is an effective strategy for achieving intellectual buy-in amongst stakeholders [47]. SMEs identified this could be facilitated by using local data, such as local costing [48], activity [49] or access data [50] that was meaningful to health administrators. While burden of disease and national or international data are compelling, where feasible, these should be supplemented by comparable data at a local level. This enabler has also been reported in other recent studies [47].

All stakeholders, perceived value in MoCs as a blueprint for guiding implementation efforts and specifically providing detail on *how* services could be delivered to a community. The use of locally derived pilot data to demonstrate proof of concept for a particular MoC was considered an important enabler in this regard. For example, the extended rollout of a system inversion MoC for pain management services in Western Australia has been facilitated by initial pilot work in program delivery [51] and aligned health workforce and consumer education [52–54]. In this context, a MoC could be considered as a knowledge translation intervention. Support for pragmatic, health services and implementation research is therefore an important consideration for those tasked with the development and implementation of MoCs.

A further identified enabler was the provision of a detailed implementation plan, or guidance on the development of a business plan. This enabler resonates, for example, with international initiatives to develop fracture liaison services for osteoporotic fractures [55]. A concerted international effort has been made to evaluate MoCs for re-fracture prevention and to provide guidance on specific implementation strategies and quality standards [56].

### Development of an evaluation framework and associated challenges

SMEs identified evaluation of MoCs as a fundamental principle, and ideally a routine practice after implementation to confirm improvements in service quality, efficiency and consumers’ access and satisfaction – arguably, elements defining ‘success’. However, the concept of an evaluation of ‘readiness’ for implementation was less familiar, yet very strongly supported, particularly in the context of assessing organisational readiness for change as one important component of an overall judgement of readiness. Notably, this was also the most significant challenge identified by the SMEs in this study. Although the state of New South Wales uses formative evaluation to examine readiness and applicability, and summative or impact evaluation to determine success, this approach is not completely standardized or nationally adopted [57].

In the business sector, organisational readiness for change is routine and well described, such as Kotter’s 8 Step model [58]. However, these concepts, skillsets and evaluation capabilities are arguably less well developed in healthcare environments, and is perhaps one factor underpinning unsuccessful implementation of MoCs [25, 59]. In one of the largest systematic reviews conducted to date on the effectiveness of chronic care models, Davy and colleagues [33] reported that no differences could be identified in health and practice outcomes between variant models. It was, therefore, impossible to identify which aspects of the chronic care models led to improvements in health outcomes and practices. They concluded that factors other than the implementation of the model *per se*, particularly organisational, personal and communication-related, have an important influence on outcomes. A recent review of organisational readiness for change in healthcare identified that psychological and structural domains were the most important domains to consider [60]. However, currently available tools to assess these domains lacked reliability and content, construct and predictive validity [60]. That review, and others [61–63], support the concept that a framework to evaluate readiness is needed, particularly as it relates to implementation of chronic disease MoCs [59], and should consider psychological and structural factors [62]. It is not surprising then, that several protocol papers to develop organisational readiness for change tools have been published recently [59, 64, 65] and importantly, reflect just one component of an overall judgement of MoC readiness.

### Strengths and limitations

This is the first study to qualitatively explore Australian stakeholders’ views on contemporary MoCs for musculoskeletal health. Strengths include purposeful sampling across multiple stakeholder groups, including consumers [41]; iterative development of an interview schedule informed by pilot testing; and data analysis by a content, as well as methods, expert. While there were clear and recurrent themes identified across stakeholders suggestive of data redundancy, a limitation, in the context of a grounded theory analytical approach, is that we did not validate our conceptualisation of MoC relevance and evaluation with a wider range of stakeholders [66]. There is also a possibility of selection bias in terms of individuals who participated in this research. For example, these individuals may have been more interested in the topic, and therefore more willing to participate. Further, our inclusion criteria and sampling strategy was established *a priori* to ensure that SMEs had a minimum level of senior, professional experience relevant to our research question and a minimum level of understanding or experience with Australian musculoskeletal MoCs in order to collect meaningful and appropriately-contextualised data. Our data, therefore, reflect the views of stakeholders with experience and a contextual background in the area and not of stakeholders who are naïve to Australian musculoskeletal MoCs. Although this design choice limits generalizability of the findings to the categories of stakeholders sampled, we do not consider this a limitation, but rather a focused design strategy to address the research question, which is an approach congruent with qualitative research principles, particularly purposive sampling [67]. Further, our purposive sampling approach ensured a spread in experiences and familiarity with the MoCs (Table 1). A limitation in our sampling approach was the absence of representation from the orthopaedic surgery discipline. In the current study, we intentionally did not aim to define the components of, or criteria for, a ‘readiness’ and ‘success’ evaluation framework, as this is the focus of ongoing research by our group.

## Conclusions

MoCs appear critical for influencing and initiating changes to best-practice healthcare delivery, and for practical guidance on how to implement and evaluate services. An evaluation framework for musculoskeletal MoCs that is particularly focused on capturing ‘readiness’ is important to ensure successful and sustainable implementation efforts. Balancing sufficient detail against an overly simplistic evaluation framework and identifying organisational readiness for change are key challenges in this regard.

## Additional file

Additional file 1:
**Consolidated criteria for reporting qualitative studies (COREQ):** **32-item checklist.** (DOCX 110 kb)

## Abbreviations

MoC: Model of care
